# Adaptation to divergent larval diets in the medfly, *Ceratitis capitata*

**DOI:** 10.1111/evo.13113

**Published:** 2016-11-24

**Authors:** Philip T. Leftwich, William J. Nash, Lucy A. Friend, Tracey Chapman

**Affiliations:** ^1^ School of Biological SciencesUniversity of East AngliaNorwich Research ParkNorwichNR4 7TJUnited Kingdom

**Keywords:** Body size, development time, developmental survival, divergence, experimental evolution, sexual selection

## Abstract

Variation in diet can influence the timing of major life‐history events and can drive population diversification and ultimately speciation. Proximate responses of life histories to diet have been well studied. However, there are scant experimental data on how organisms adapt to divergent diets over the longer term. We focused on this omission by testing the responses of a global pest, the Mediterranean fruitfly, to divergent selection on larval diets of different nutritional profiles. Tests conducted before and after 30 generations of nutritional selection revealed a complex interplay between the effects of novel larval dietary conditions on both plastic and evolved responses. There were proximate‐only responses to the larval diet in adult male courtship and the frequency of copulation. Males on higher calorie larval diets consistently engaged in more bouts of energetic courtship. In contrast, following selection, larval development time, and egg to adult survival showed evidence of evolved divergence between diet regimes. Adult body size showed evidence for adaptation, with flies being significantly heavier when reared on their “own” diet. The results show the multifaceted responses of individuals to dietary selection and are important in understanding the extreme generalism exhibited by the medfly.

The responses of individuals to dietary nutrients represent an important driver for natural selection (Raubenheimer et al. 2009). The combinations of nutrients that organisms gain from their diets are essential for all aspects of life histories, including development and sexual competitiveness (Stearns 1992). Quantitative and qualitative variations in dietary nutrients significantly influence the timing of major life history events such as reproduction (Simpson and Raubenheimer 1993) and can direct allocation decisions and trade‐offs (Stearns 1992). Divergent selection mediated by differing diets is also important in driving population diversification and speciation (e.g., Coyne and Orr 2004; Nosil 2012). A large body of research has revealed the proximate responses of life histories to diet (Maor et al. 2004; Romanyukha et al. 2004; Davies et al. 2005) and the mechanistic underpinnings involved (e.g., the insulin and rapamycin pathways; Neufeld 2004; Partridge et al. 2011). In invertebrates the importance of nutrients to survival, growth, courtship, mate selection, fertility, reproductive success, and lifespan have been particularly well studied (e.g., Chapman and Partridge 1996; Yuval et al. 1998; Kaspi et al. 2002; Dmitriew and Rowe 2011; Vijendravarma et al. 2012).

In insects, diet quality and/or quantity is of major importance during both developmental and adult stages of the life history. Carry‐over effects of nutrition from development to adulthood are also reported to influence reproduction, aging, and tolerances to stress (Yuval et al. 1998; Tu and Tatar 2003; Hahn 2005; Andersen et al. 2010; Dmitriew and Rowe 2011). The acquisition of nutrients during larval feeding supports immediate growth, but also future investment, providing resources that are subsequently utilized by the pupa and adult. For almost all holometabolous insects, growth during the larval phase, as well as the accumulation of nutritional reserves, are vital for the survival of the nonfeeding pupal stage and often represent a significant fraction of the adult energy budget (e.g., Vijendravarma et al. 2012; Chapman et al. 2013). Many holometabolous insects have the ability to accumulate resources as larvae that augment or supplement adult nutrient intake and hence enhance reproductive success of adults of both sexes (De Block and Stoks 2005; Pechenik 2006).

Given the importance of the larval growth phase for setting adult body size and energy budgets, robust mechanisms have evolved to ensure that larvae develop toward a physiologically determined set point referred to as the “critical weight” before the onset of metamorphosis (Davidowitz et al. 2003; Nijhout 2003a; Davidowitz et al. 2005). Larvae show a robust ability to alter growth rates in response to differing diets through changes in the expression of the endocrine system. For example, in *Drosophila* the insulin receptor (InR) cascade can influence the speed of larval development prior to the critical weight threshold (Mirth et al. 2005).

It is also becoming increasingly clear that plasticity in dietary responses is a crucially important determinant of trade‐offs and interactions with environmental factors. These responses can play an important role in driving evolutionary change (West‐Eberhard 2003; Levis and Pfennig 2016). For example, the adaptive flexibility of an organism to its environment is predicted to facilitate the origin of novel morphological and behavioral features. Ultimately, this may serve as a first step in the process of adaptive evolution. However, the relative importance, and temporal influence, of phenotypic plasticity to divergence is currently a topic of much debate. The identification of variable versus fixed responses can be challenging (Levis and Pfennig 2016). Part of the problem in studying the nature of evolved responses is the lack of evolutionary experiments in which the initial stages of divergence can be observed in real time. Here, we addressed this omission by conducting experimental evolution upon divergent larval diets in the Mediterranean fruitfly (medfly, *Ceratitis capitata*).

The medfly is highly suitable for such studies. It is experimentally tractable and of key applied importance as it exploits a diverse and expansive range of larval hosts under natural conditions. Understanding how it does so is key to understanding its invasive potential (Diamantidis et al. 2011). The medfly has become a globally distributed pest over the past ∼150 years and is capable of causing extensive damage to a wide range of commercially important fruit crops. It exhibits extensive plasticity in host selection, utilization, and egg laying behavior (Féron 1962; McDonald and McInnis 1985; Katsoyannos 1989; Yuval and Hendrichs 2000) and has an exceptionally wide host range of >350 species (Liquido 1991). Larvae can successfully complete development within a wide range of economically important fruits (Carey 1984) and this developmental plasticity is also maintained in the laboratory (Nash and Chapman 2014). The growth and development of a thriving international fruit trade has led to the medfly becoming a dangerously invasive pest of global economic importance (Siebert and Cooper 1995; Siebert 1999; Papadopoulos 2014).

As a result of the development of mass rearing strategies for insect control programmes (SIT (Knipling 1955; McInnis et al. 2002) and RIDL (Alphey et al. 2008; Leftwich et al. 2014)) there has been extensive investigation into the relative importance of the larval diet in determining growth, development, and adult mating success in medfly. For example, many studies have also sought to use dietary interventions to maximize the productivity and sexual performance of medfly populations subject to mass release control programmes (McInnis et al. 2002; Lance 2014). Such studies have highlighted that the medfly is capable of considerable plasticity both in larval development and behavior (e.g., migration to those areas within a host of highest nutritional quality). This permits the use of a range of larval diets and broadens the potential for the utilization of a range of larval hosts. At the same time, experimental manipulation of carbohydrate and protein levels in homogenous environments can drastically impact on larval survival, development, and adult body size (Nash and Chapman 2014).

Interestingly, geographically isolated populations of medfly exhibit significant fixed differences in pre‐adult development rates and survival when placed on common garden diets (Diamantidis et al. 2008). This may affect their invasive potential, but it is unclear what mechanisms drive these changes. Local adaptation to dietary conditions may impact on the invasive potential of the Tephritid species such as the medfly (Godefroid et al. 2015) and, as gene flow between global populations of medfly is becoming increasingly reduced (Karsten et al. 2015), this potential for localized adaptation may increase. Hence population‐specific control measures may increasingly be required.

The collective body of research described above emphasizes the need to understand medfly adaptation in a nutritional context to facilitate biotechnological advances for control (Scolari et al. 2014). However, while there is an existing body of literature on proximate responses to dietary interventions, there is a lack of knowledge of how medfly evolves in response to different diets over the longer term. The evidence of divergent physiological traits among isolated medfly populations (Diamantidis et al. 2011) is likely to be influenced by adaptation to different host nutritional profiles, but this is as yet unproven. Such knowledge would also be relevant to understanding how the adaptive flexibility of generalist species is affected over multiple generations of consistent selection where gene flow is curtailed.

We addressed the lack of knowledge of dietary adaptation in medfly in this study by conducting experimental evolution using divergent larval diets and measuring developmental and adult correlates of fitness under both dietary regimes. We measured the effect of altered diet components on pre‐adult development time and survival rates and used measures of adult mating rates and courtship behaviors to assess the carryover effects of larval diet to adult reproductive fitness.

## Materials and Methods

We conducted replicated experimental evolution under two divergent larval diet regimes: ASG, “A” (a high calorie diet with a mix of simple and complex carbohydrates) and Starch, “S” (a lower calorie diet comprising complex carbohydrates) (details below). The diets were chosen to provide qualitative and quantitative variation in calories, and, as our previous work showed, both diets were able to successfully support larval development (Nash and Chapman 2014). We assayed developmental characteristics at generations 3–5 and 30 of the experimental evolution, along with courtship behavior tests at generation 30. Tests were conducted for both regimes on both diets to create four different treatments (parental food/focal food: A/A, S/S or A/S, S/A). To test the responses of medfly to their proximate larval diet versus the larval diet to which they have adapted over many generations, development time and survival, body mass, and courtship behavior were measured.

### FLY STOCKS AND CULTURING

The base stock from which the experimental evolution lines were derived was the TOLIMAN strain originating from Guatemala, which has been reared in the laboratory since 1990 (Morrison et al. 2009). For at least two years prior to the start of these experiments TOLIMAN was reared on a wheat bran diet (24% wheat bran, 16% sugar, 8% yeast, 0.6% citric acid, 0.5% sodium benzoate). To initiate the experimental evolution, flies from the TOLIMAN stock population were established on two larval diets, (i) sucrose‐based “ASG” (A) medium (1% agar, 7.4% sugar, 6.7% maize, 4.75% yeast, 2.5% Nipagin (10% in ethanol), 0.2% propionic acid; 684kcal/L) or (ii) “Starch” (S) medium (1.5% agar, 3% starch, 5% yeast, 0.5% propionic acid; 291 kcal/L). The caloric value of both larval diets was estimated from published sources, with the ASG diet estimated to comprise roughly twice the amount of available Kcal/L (Southgate and Durnin 1970; USDA 2015). Three independent biological replicates of each regime were maintained under strict allopatric conditions. All experiments and culturing were conducted at 25°C, 50% relative humidity, on a 12:12 light dark photoperiod. Adults emerging from each replicate were maintained in groups of roughly 30 males and 30 females in plastic cages (11 cm × 11cm × 10 cm). Adults from all lines received the same standard adult diet (ad libitum access to sucrose‐yeast food; 3:1 w/w yeast hydrolysate/sugar in water). Each generation, approximately 500 eggs were placed on 100 mL of the appropriate diet in a glass bottle. When third instar larvae started to “jump” from the larval medium, the bottles were laid horizontally on sand and pupae allowed to emerge for seven days. Pupae were then sieved from the sand and held in 9 mm petri dishes until adult eclosion.

### DEVELOPMENTAL ASSAYS

#### Egg to adult survival

To test for evidence of divergence or dietary adaptation (i.e., differences between selection regimes, vs increased performance under “own” as opposed to “opposite” diet conditions, respectively), each of the three independent biological replicates for each of the two dietary regimes were tested at early and late time points (generations 3–5 and 30, respectively). Flies were tested on their own regime larval diet and by crossing onto the opposite larval diets for two generations, to differentiate selection effects from proximate diet or parental effects. At generation 3 flies were reared on their own larval food regime for testing (i.e., regime food/test food: A/A *N* = 6000 and S/S treatments *N* = 6000). For testing at generation five, flies were reared on the opposite diet (i.e., regime food/test food: A/S N = 6000, S/A treatments N = 6000). At generation 30, all four treatments (A/A, S/S, A/S, S/A N = 5400 per treatment) were tested simultaneously.

Eggs were collected over a 24‐h period and counted under a dissecting microscope. Eggs were then incubated on wet Whatman filter paper (Fisher Scientific) and sealed within ten Petri dishes each containing either 40 g of own versus opposite larval food medium (ASG or Starch, 0.2 g/egg, 200 eggs per Petri dish, 2000 eggs per line in total). When third instar larvae started to “jump” from the larval medium, the plates were unsealed and laid on sand and pupae allowed to emerge for seven days. Each plate was checked daily and the number of new pupae formed was recorded. Pupae from each line were kept and monitored for eclosion, and adults were checked for sex before recording the day of eclosion. Noneclosed or partially eclosed pupae casings were counted and then discarded.

#### DEVELOPMENT TIME

Development was recorded as the median time (in days) from egg collection to pupation and adult eclosion for each Petri dish. To measure the effect of larval diet and experimental adaptation on body mass, the dry weights of males and females from the development tests were taken by freezing individuals posteclosion at –20°C for 24 h, followed by desiccation at 25°C for 24 h and weighing samples of 100 flies from each replicate/treatment on a BDH DE‐100A micro‐balance.

### VIDEO DATA ANALYSIS OF MALE COURTSHIP BEHAVIOR

Courtship behavior was analyzed during the 29th and 30th generation of experimental evolution. The treatments comprised of single pairs (one male, one female) in a fully factorial design. In generation 29 “own diet” females and “own diet” males were tested, in generation 30 “own diet” females and “crossed diet” (opposite diet) males. Flies were reared in single‐sex cages as above until the 7th day posteclosion, when mating tests were conducted. At lights on (09.00) females were aspirated into mating arenas (50 mm × 11 mm Petri dishes) 30 minutes prior to the introduction of a male. Each arena had 10 mm × 30 mm strip of paper tape added to the outer lid surface to simulate the underside of a leaf and facilitate normal courtship behavior. Observations began with the introduction of a male and continued until for 30 minutes or until a successful copulation occurred. Filming was conducted under ambient lighting using Sony Handycam CX190 high definition video cameras. An adjustable shelving unit was used to suspend the filming cells approximately 20 cm above the cameras.

### BEHAVIORAL QUANTIFICATION

Based on preliminary analyses and previous studies (Briceño et al. 1996; Briceño and Eberhard 2002; Briceño et al. 2007) six behaviors were selected to quantify Medfly courtship (Table 1). Mutually exclusive and nonmutually exclusive (co‐occurring behaviors) were scored.

**Table 1:** Description of precopulatory behaviors in the analysis of male courtship

Behavior	Description
Gland out	Focal male extrudes anal pheromone gland (not associated with preening)
Continuous wing buzzing	Focal male buzzes wings continuously
Head rock	Focal male moves head from side to side rapidly
Intermittent wing buzzing	Focal male buzzes wings, while flapping them rapidly
Copulation	Focal male achieves intromission of genitals
Copulation attempt	Focal male attempts intromission of genitals but is dislodged by female

### STATISTICAL ANALYSIS

All data analysis was conducted in R v3.2.0. (R Development Core Team 2015) using the “lme4” (Bates et al. 2015), “lmertest” (Kuznetsova 2016), “multcomp” (Hothorn 2008) and “MuMIn” (Bartoń 2013) packages.

#### Development

Developmental survival was treated as proportion data calculated from the number of individuals that entered a development stage versus those that completed it. These data were analyzed by generalized linear‐mixed models (GLMMs) using a binomial distribution. Observation‐level random effects were employed to account for data that were overdispersed (identified by comparison of the residual deviance with the residual degrees of freedom). Models that encountered convergence errors were fitted with the “bobyqa” optimizer. Development time was measured as the median number of days (to the nearest 12 h) for flies from a cohort to reach each discrete developmental stage and analyzed by using linear mixed models (LMM). Body mass was the dry weight of males and females analyzed by LMM. Data from early and late generations were analyzed together with generations, selection regime, and proximate larval diet designated as discrete factors and included in the models as fixed effects. Replicate lines were nested as a random effect within selection regime. These data were then split and the dietary responses of flies at early versus late generations were analyzed separately. Initial models included all possible interactions and sequential model selection was conducted by likelihood ratio testing, using lmertest: ANOVA. After each model of developmental data was fitted, a marginal *r^2^* value was calculated to express the variance explained by the fixed factors using “MuMIn.” Significance of treatment comparisons was assessed using Tukey HSD multiple comparison tests using the “glht” function within “multcomp.”

#### Male courtship and copulation behavior

Video data were analyzed using VLC media player. Each video was scored for male behaviors using JWatcher ver. 1.0 (Blumstein et al. 2006) using a double blind procedure to minimize observer bias. Behaviors were scored sequentially and categorized as behavioral states or elementary behaviors using the JWatcher focal analysis master file function.

We calculated two metrics. Bout Frequency (BF) was the number of times a specific behavior occurred from the start of filming to the occurrence of copulation. Total time spent in a behavior (TT) was the sum of the durations of all bouts of a specific behavior, in milliseconds, from the start of filming to the occurrence of copulation. Latency to the initiation of courtship and to successful copulation was also scored. Latency to courtship was the time in milliseconds from the initiation of the mating test to the first occurrence of one of the four courtship behaviors (Table 1). Latency to copulation was the time in milliseconds from the initiation of the mating test to the observation of a settled copulation. GLMMs as previously described were fitted for the bout frequencies (BF) and total number of milliseconds (TT) for which each behavior occurred. Models were offset to the log of the duration until copulation, to account for differing lengths of overall courtship time.

#### No‐choice mating tests

The success of males in securing copulations during 30‐minute no choice mating tests was recorded as success or failure, and analyzed using a Chi‐Square test for equality of proportions (Wilson 1927).

## Results

#### Egg to adult survival

**Figure 1:**
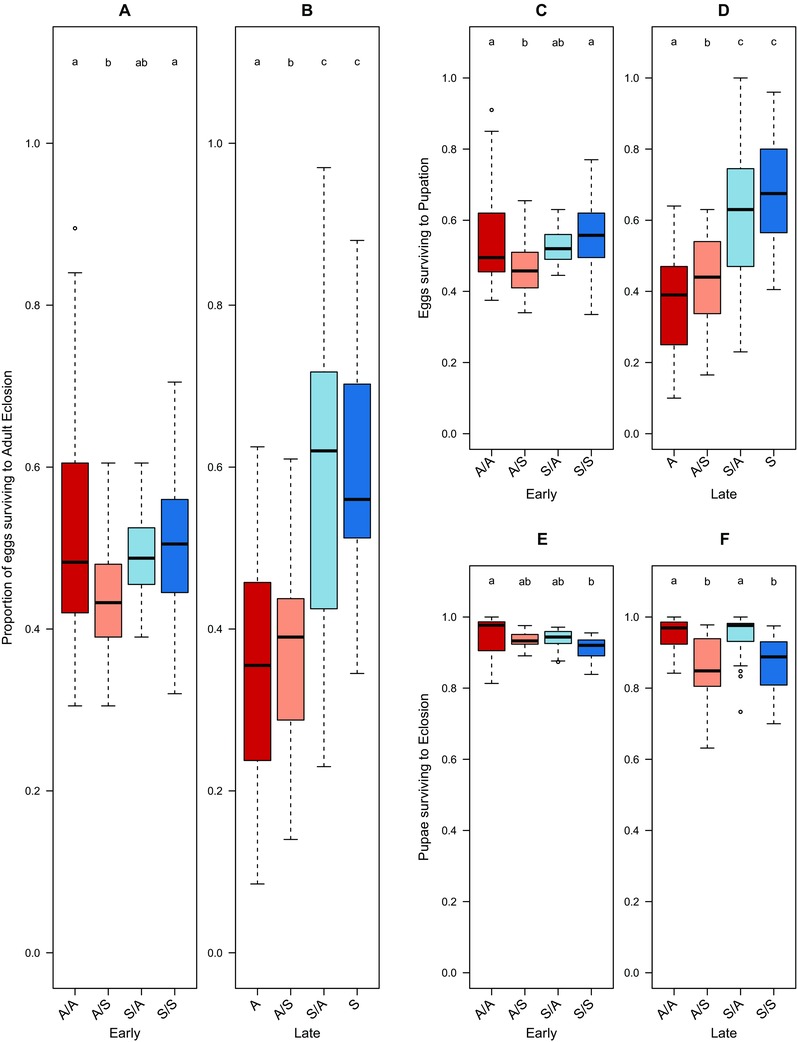
Proportion of Medfly individuals surviving between each developmental stage at generations 5 and 30 of artifical selection on divergent larval diets. Developmental survival of individuals derived from ASG (A) or Starch (S) larval dietary regimes and maintained/crossed to proximate larval diets of either ASG or Starch (parental food/focal food: A/A, S/S or A/S, S/A). Left hand panel of each pair = early (five generations) and RH panel late (30) generations of selection. Panels A, B show egg to adult survival; panels C, D larval survival (proportion of eggs surviving to pupation); panels E, F (pupal survival (proportion of pupae surviving to adult eclosion). Lower case letter groupings denote significant differences at P < 0.05 following posthoc analysis.

We found that the number of individuals surviving development from egg to adult eclosion was affected by a significant interaction between selection regime and generation, but with no effect of proximate larval diet (glmer; generation × regime; *z* = 6.45, *P* < 0.001, *r^2^* = 0.03, Table S1A, Fig. 1A, B). For the early generation data, we found a significant effect of regime by proximate larval diet on developmental survival (glmer; regime × diet; *z* = 3.44, *P* < 0.001, *r^2^* < 0.01; Fig. 1A, Table S1B). Individuals from the ASG and Starch regimes reared on their own regimes did not differ in egg to adult survival. Flies switched from ASG to Starch had significantly lower survival, which was not evident in the reciprocal swap (Starch to ASG). At generation 30 (Fig. 1B), the regime from which the flies were derived was the only significant predictor of developmental mortality (glmer; regime; *z* = 7.58, *P* < 0.001, *r^2^* = 0.07; Table S1C). The survival of late generation individuals from the Starch regimes was unaffected by proximate larval diet, and was higher than for the ASG selected individuals.

#### Larval survival

Larval survival (i.e., number of eggs that reached the start of pupariation) was broadly consistent with the overall patterns of egg to adult mortality described above (Fig. 1C, D). Survival from egg to pupation was influenced by two sets of interactions (glmer; generation × regime; *z* = 6.59, *P* < 0.001; generation × diet; *z* = 2.47, *P* = 0.014, *r^2^* = 0.05; Table S1D). When these data were split, and analyzed using separate models for early and late generation effects, the early generation data showed a significant interaction effect of selection regime and proximate larval diet (*z* = 3.81, *P* < 0.001, *r^2^* < 0.01; Table S1E). Switching flies from ASG to Starch media produced a significant decrease in larval survival; this was not evident when switching flies from Starch to ASG (Fig. 1C). By contrast, the generation 30 data indicated that selection regime was the only significant predictor of larval survival (Fig. 1D), with no effect of the proximate larval diet (glmer; regime; *z* = 7.68, *P* < 0.001, *r^2^* = 0.08, Table S1F). The larval survival of late generation individuals from the Starch regimes was unaffected by proximate larval diet and was higher than for ASG selected flies.

#### Pupal survival

Pupal survival (number of pupae that became adults) was affected by interactions of generation with both regime and with proximate diet (glmer; generation × regime; *z* = 2.12, *P* = 0.028; generation × diet; *z* = –3.73, *P* < 0.001, *r^2^* = 0.06; Table S1G). In contrast to larval survival, when the data were split across early and late generations, pupariation survival was high and invariant between regimes. Any differences were primarily due to proximate diet (glmer; generation 5 diet; *z* = –3.82, *P* < 0.001, *r^2^* = 0.01, Table S1H; generation 30 diet; *z* = –6.96, *P* < 0.001, *r^2^* = 0.09; Table S1I). At generation 5, flies from the Starch regime reared on starch had reduced pupal survival, flies from the ASG regime had the highest pupal survival, while both diet switched treatments had intermediate survival proportions (Fig. 1E). By generation 30 there was a clear pattern of reduced pupal survival on a proximate Starch diet regardless of selection regime (Fig. 1F).

These analyses gave evidence for a dietary divergence between the regimes at the later generations, with Starch lines having higher survival than ASG, regardless of the proximate diet on which they were reared (Fig. 1B). During the early generations of selection, proximate larval diet was the main predictor of survival. However, after 30 generations, the ASG and Starch regimes showed significant and repeatable divergences in survival regardless of immediate larval diet.

#### Egg to adult development time

**Figure 2:**
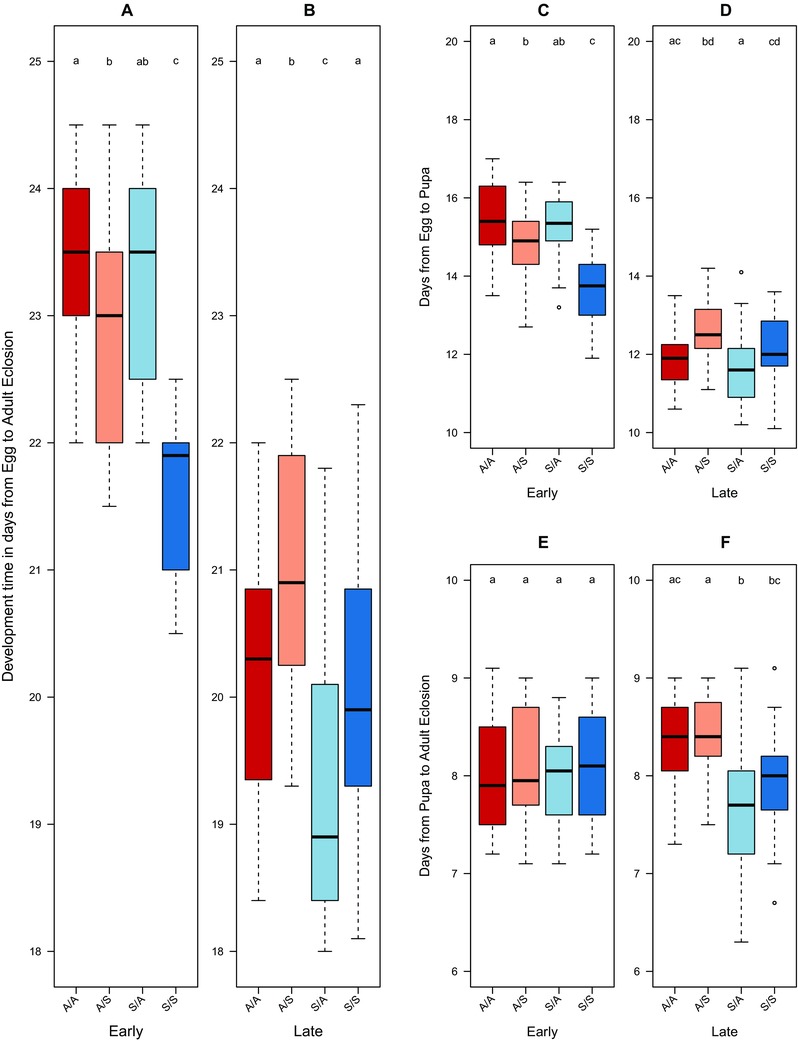
Time (in days) for Medfly individuals to complete each developmental stage at generations 5 and 30 of artifical selection on divergent larval diets. Development time for individuals derived from ASG (A) or Starch (S) larval dietary regimes and maintained/crossed to proximate larval diets of either ASG or Starch (parental food/focal food: A/A, S/S or A/S, S/A). Left hand panel of each pair = early (five generations) and RH panel late (30) generations of selection. Panels A, B show egg to adult development times, panels C, D development time from egg to pupation, panels E, F development time from pupa to adult eclosion. Lower case letter groupings denote significant differences at P < 0.05 following posthoc analysis.

Overall development time was significantly influenced by interactions between regime × proximate diet and by generation × proximate diet (lmer; regime × diet; *t_228_* = –2.41, *P* = 0.017, generation × diet; *t_228_* = 7.8, *P* < 0.001, *r^2^* = 0.73; Table S2A). In the early generations, the speed of development was determined by an interaction of regime and proximate larval diet (lmer; regime × diet; *t_120_* = –3.65, *P* < 0.001, Table S2B, *r^2^* = 0.44; Fig. 2A), with flies on a proximate starch diet showing a significantly shorter development time (Fig. 2A). In generation 30, egg to adult development appeared to have reduced in comparison to the early time point for all treatments (lmer; regime; *t_108_* = –4.34, *P* < 0.001; diet; *t_108_* = 4.21, *P* < 0.001, Table S2C, *r^2^* = 0.25; Fig. 2B).

#### Larval development time

Larval development time (from egg to pupariation) was significantly affected by interactions between regime × proximate diet and regime × generation (lmer; regime × diet; *t_228_* = –2.76, *P* = 0.006; generation × diet; *t_228_* = 8.1, *P* < 0.001, *r^2^* = 0.75; Table S2D). During the early generations, larval development time was significantly influenced by regime and proximate larval diet, with the fastest development being observed in flies from the Starch regime reared on the Starch diet (S/S) (lmer; regime; *t_120_* = –4.16, *P* < 0.001; diet; *t_120_* = –6.92, *P* < 0.001, *r^2^* = 0.35; Table S2C). However, at generation 30, larval development was faster across all treatments (Fig. 2C, D) and influenced only by proximate diet, with flies reared on Starch (A/S and S/S) developing more slowly than those reared on ASG (lmer; diet *t_102_* = 4.36, *P* < 0.001, *r^2^* = 0.14; Table S2F).

#### Pupal development time

The time spent in pupal development was significantly influenced by the regime × generation interaction (lmer; generation × regime *t_228_* = –3.69, *P* < 0.001, *r^2^* = 0.12; Table S2G*)*. When these data were split by generations it became apparent that the model fit was poor for the early generation data, with no significant predictors of pupal development time (Fig. 2E). However, in later generations there was evidence of divergence (Fig. 2F), with the ASG regime flies having significantly longer pupal development, regardless of proximate diet (lmer; regime *t_108_* = –5.01, *P* < 0.001, *r^2^* = 0.19; Table S2H).

After 30 generations of selection, development time had decreased and the regimes showed evidence of divergent responses to diet. The overall pattern of egg to adult development time was largely determined by larval to pupariation duration. Pupal development time showed less variation across regimes or diets.

#### Adult body mass

**Figure 3:**
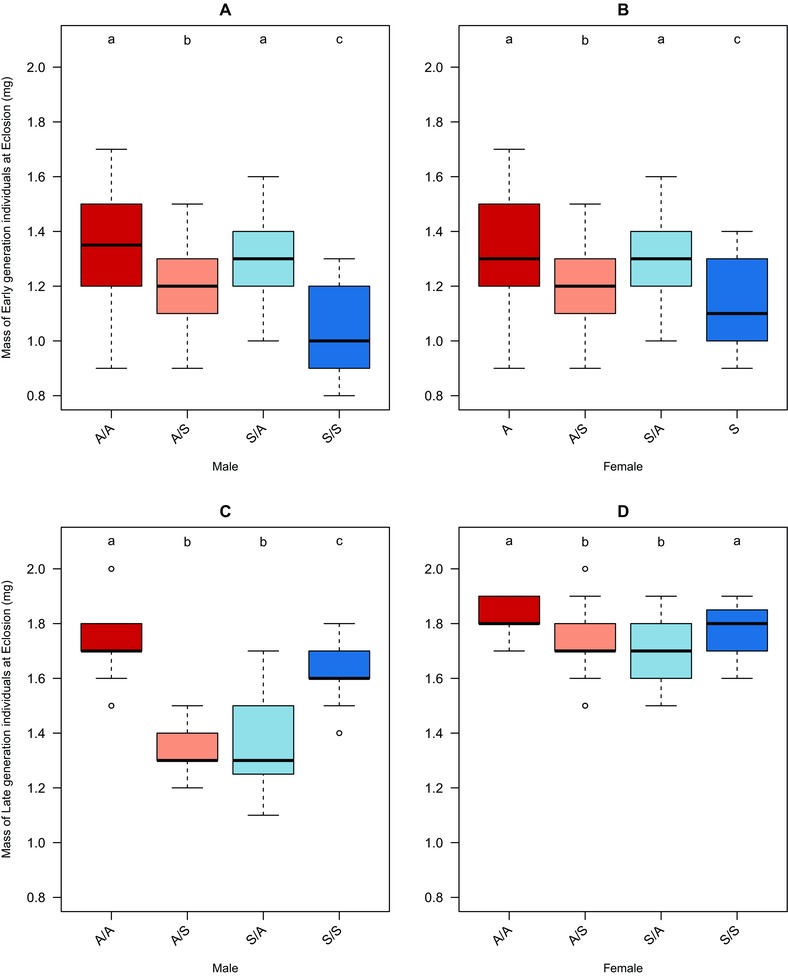
Mass at eclosion of Medfly males and females at generations 5 and 30 of artificial selection on divergent larval diets. Mass at eclosion of individuals derived from ASG (A) or Starch (S) larval dietary regimes and maintained/crossed to proximate larval diets of either ASG or Starch (parental food/focal food: A/A, S/S or A/S, S/A). Top row = early (five generations), panel A shows mass at eclosion for males, B shows mass at eclosion for females. Bottom row = late (30) generations of selection. Panel C shows mass at eclosion for males, D shows mass at eclosion for females. Lower case letter groupings denote significant differences at *P* < 0.05 following posthoc analysis.

Adult male body mass was predicted by a three‐way interaction of proximate diet, selection regime, and generation (lmer; *t_228_* = 6.25, *P* < 0.001, *r^2^* = 0.67; Table S3A). In contrast, female body mass was determined by an interaction of diet and generation, with regime as a significant main effect (lmer; generation × diet, *t_228_* = 4.19, *P* < 0.001; regime, *t_228_* = –3.27, *P* = 0.001, *r^2^* = 0.69; Table S3B). In the early generations there was a consistent male and female body mass advantage in response to rearing on the ASG larval diet (Fig. 3A, B). Interestingly, there was a significant effect of both regime and proximate diet for both sexes and the body weight distributions of both sexes by treatment were similar (lmer; males; regime × diet, *t_120_* = –3, *P* = 0.003, *r^2^* = 0.24, Table S3C; lmer; females; regime, *t_120_* = –4.35, *P* < 0.001; diet, *t_120_* = –3.92, *P* < 0.001; *r* ^2^ = 0.22, Table S3D). ASG regime individuals reared on Starch had a significantly greater bodyweight than Starch regime individuals (Fig. 3A, B). In the late generation, there was evidence in males and females from both regimes for adaptation to their respective diets, with an increase in adult bodyweight on their own versus opposite diets, in comparison to the early generation (Fig. 3C, D). This is consistent with the idea of nutritional adaptation of each line to its own specific diet. The effect was significant in both sexes, but was more pronounced in males (lmer; males; regime × diet interaction *t_108_* = 6.35, *P* < 0.001, *r^2^* = 0.3, Table S3E; lmer; females, diet; *t_108_* = 2.08, *P* = 0.04; Table S3F, *r^2^* = 0.04).

During the early generations of selection there was an observed trend toward greater body mass by males and females when reared on a proximate ASG larval diet. The later generations showed a strong signal of adaptation, as both sexes demonstrated significantly higher adult body mass when reared on their own larval diets (Fig. 3C, D).

#### No‐choice mating tests

The results of the no choice mating tests (Table 2) showed that in generation 29, there were significant differences between treatments in the proportions of “on (own) diet” males that successfully copulated *Χ^2^_3_* = 38.96, *P* < 0.001). ASG males mated significantly more frequently than did Starch males. This pattern was repeated with ASG females, with ASG:ASG pairings being the most frequent (Table 2). In generation 30, when males were on crossed (opposite) diets and paired with uncrossed (own diet) females, there were significant differences between treatments (*Χ^2^_3_* = 24.84, *P* < 0.001). ASG males reared on Starch were significantly less likely, and Starch females more likely, to be observed in mating pairs. The most frequently observed mating pair was S/A males with Starch females (Table 2).

**Table 2:** Mating test results of no choice mating tests conducted during the 29th and 30th generations of the evolution experiment

Generation	Male background	Female background	Total mating tests	Total copulations	Percentage of copulations	Sample size of individual pairs subjected to behavioral analysis
29	ASG	ASG	78	58	74%	50
		Starch	75	42	56%	35
	Starch	ASG	59	26	44%	22
		Starch	62	14	23%	12
30	ASG on Starch	ASG	60	12	20%	9
		Starch	67	23	34%	19
	Starch on ASG	ASG	56	21	38%	19
		Starch	58	37	64%	33

The percentage and sample size of copulations recorded and analyzed for each pair type is presented.

Overall the pattern of results suggested a strong effect of proximate larval diet on male courtship vigour.

#### Courtship and copulation latency

There was a significant effect of dietary background on courtship latency for males and females reared on “own diet” (Gen 29, glmer; males, *z* = 2.53, *P* = 0.012, females, *z* = 3.15, *P* = 0.002, Table S4A). ASG males were significantly faster to initiate courtship than were Starch males. There was also a significant effect of male diet on the latency to successful copulation (glmer; *z* = 3, *P* = 0.003, Table S4B), with ASG males being faster at securing copulations regardless of female background. When males were placed onto a switched diet (Gen 30), there were no significant effects of male or female dietary background on either courtship or copulation latencies.

#### Courtship behavior

**Figure 4:**
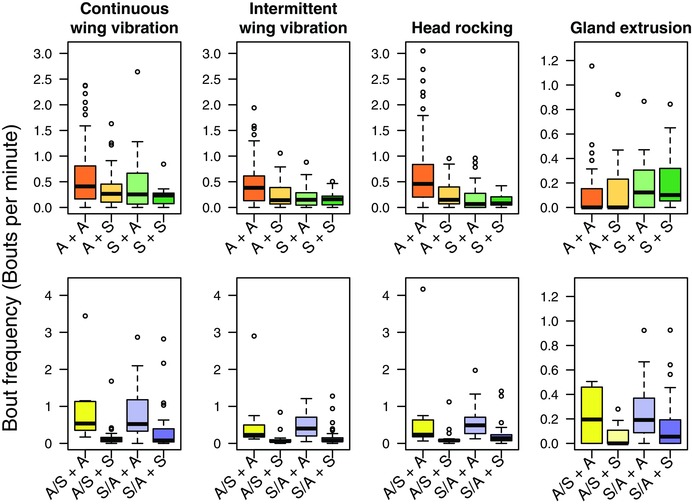
Bout frequency of courtship behaviors in generation 29 and 30. Major courtship behaviors occurring in pairs of males/females. Top row shows males reared on “own diet” (A or S) (tests conducted at gen 29), the bottom row males reciprocally crossed onto the opposite diet (A/S or S/A, tested at generation 30). Each male was paired with a single female (A or S, i.e. from either the ASG or Starch regime – all females being reared on their “own” diet). Bout frequency was scaled by copulation latency for each individual to give “bouts per minute of behavior.”

When males were reared on “own diet” (Gen 29), their dietary background had a significant effect on the number of bouts of all recorded courtship behaviors (glmer; *P* < 0.05 in all cases, Fig. 4, Table S5A–D). ASG males conducted significantly more bouts of continuous and intermittent wing vibrations and head rocking, while Starch males conducted significantly more bouts of gland extrusion (Fig. 4). Males from both diet backgrounds conducted significantly more bouts of all courtship behaviors when paired with an ASG female (glmer, *P* < 0.02 in all cases, Table S5A–D). On “diet crossed” male treatments (Gen 30), there was no significant effect of male dietary background on courtship behaviours. However, male courtship behaviors were significantly affected by female dietary background, ASG females elicited more bouts of all courtship behaviors from all males (glmer, *P* < 0.004 in all cases, Fig. 4, Table S5E–H).

Following the sequential analysis of courtship behavior described above, a pattern of activity consistent with the copulatory success described in Table 2 was observed. Males selected on the ASG diet initiated courtship faster than Starch males, secured copulations earlier and also conducted significantly more courtship behavior. Starch males conducted more bouts of gland extrusion. The background of the female also significantly affected behavior, ASG females appeared to elicit more courtship than did Starch females, as well as more noncourting “orientation” behavior.

## Discussion

The results revealed a pattern of plastic and evolved responses to larval dietary selection in the medfly. We observed traits that responded primarily to immediate nutrient availability (“proximate” effects) and also those that responded strongly to selection regime effects, showing evidence for divergent, and in one case adaptive, responses.

One of the most interesting results was that adult body mass showed evidence of adaptation over time, with individuals being heavier when reared on their own regime as opposed to the opposite regime at the later generation tested. The pattern of responses for egg to adult development time and egg to adult survival at the late generation showed evidence for divergence between the different nutritional selection regimes that was insensitive to proximate diet effects.

Changes in the proximate larval diet primarily affected adult male courtship and the frequency of copulation. Those males reared on the calorie rich ASG diet engaged in more bouts of energetic courtship behaviors and secured more copulations. Starch‐reared males spent more time in “passive” courtship. This result is consistent with the finding that available nutritional resources in the larval stage are of vital importance to the energy budget of sexually mature adults (Kaspi et al. 2002; Nijhout 2003a,b).

Sequential analysis of courtship behavior leading to successful copulation showed that males reared on ASG (especially those also selected on ASG) engaged in more bouts of “active” courtship behaviors (i.e., wing vibration and head rocking) and gained significantly more copulations. In contrast males reared on Starch exhibited more “passive” behaviors (such as gland extrusion (Briceño et al. 1996)) and were less likely to successfully copulate with females from either regime.

The manifestation of a more “active” courtship profile in males reared on the highly calorific ASG diet may suggest that this larval diet allowed males to store more nutrients during development (Chippindale et al. 1997). However, when individuals were “diet crossed,” the patterns of behavior leading to successful copulation did not persist. Therefore, there was no evidence of evolved differences in courtship behavior. Diet crossed males of both backgrounds expressed more of all four courtship behaviors when paired with ASG females. Such females may have been perceived as “higher quality,” perhaps associated with their rearing on the higher quality ASG diet. This is consistent with the findings that female medfly with access to higher levels of protein and sugar during development are more likely to mate, are more fecund, and reach sexual maturation more rapidly (Kaspi et al. 2002).

The observation that diet crossed males from both backgrounds elevated their level of courtship toward higher quality females, regardless of their source population, showed that there was no divergence in mate choice by regime. However, it is possible that our experimental design (i.e., no‐choice mating assays) might minimize detection of reproductive isolation. This is because this design emphasises measures of acceptance thresholds for a single female with a single male, rather than signals of preference for females that have a choice of more than one male (Edward 2014). The frequency and time spent in courtship behaviors performed toward Starch females was significantly lower by both backgrounds of diet crossed males, suggesting males perceived them as low quality mates. Despite this, Starch females were more likely to form pairs with either type of crossed male than ASG females. This suggests that the “low quality” of these females may have reduced their ability to resist male attempts to copulate, a trait important in determining copulation success in the medfly (Arita and Kaneshiro 1988; Whittier et al. 1994). Large body size, resulting from favourable developmental conditions, has also been shown to lower a female's ability to resist copulation attempts (Taylor and Yuval 1999). However, as there was little difference in female body mass, variation in female resistance is expected to be minimal. The observed difference in mating pairs containing starch females and starch males suggests that female choice, notoriously difficult to quantify (Chenoweth and Blows 2006), may play a greater role in the outcome of copulation success than previously suspected in this species.

The initial advantage of greater adult body mass for males and females reared on ASG may have been due to the greater calorific content of this diet. As selection proceeded the proximate response of body size to diet changed significantly and instead we observed evidence for adaptation. Both males and females had significantly higher body mass on their own versus the “crossed” diets. Hence, despite the capacity to express considerable plasticity, the flies adapted to best exploit the diet on which they were selected. In terms of the wider significance, should body mass represent a key fitness trait related to invasiveness, then such adaptation, if it occurs in the field, could limit the ability of the medfly to colonise new hosts (Diamantidis et al. 2011). Field studies of the extent of adaptation of medfly populations to their local hosts would be very useful in this context.

Despite the Starch medium representing a potentially poorer quality diet in terms of calories and nutritional complexity, survival to adulthood on both diets was not initially significantly different between regimes. The proportion of individuals surviving larval development diverged significantly (and consistently among biological replicates) between selection regimes over time, with flies from the ASG regime exhibiting higher larval mortality regardless of proximate diet. We observed a consistent reduction in development time from the early to late generations tested. Each regime demonstrated divergent responses to “diet crossing.” ASG regime flies were consistently slower to develop than were Starch flies, but when switched onto Starch, development was delayed still further. The opposite was true for Starch flies, which accelerated development when reared on ASG food. The pattern of results suggests that selection under the nutritional conditions experienced by each line shaped larval survival and development. Each regime appeared to evolve a characteristic and relatively fixed expression of traits, suggesting early versus late life fitness trade‐offs between the regimes, which were not affected by the proximate diets tested. ASG flies showed delayed development and higher larval mortality in comparison to Starch flies, even though they had access to a potentially more nutritious diet. They also showed higher mating propensity. We cannot rule out the effects of genetic drift. However, the replicated responses observed support the interpretation that the lines responded to selection. It would be interesting to measure the advantages of these specific traits expressing relatively invariant responses following nutritional selection. It would also be worthwhile testing further the potential impact of larval substrate trade‐offs in fitness and fully quantifying adult fitness outside of copulatory behavior (i.e., sperm quality, female fecundity, longevity). Divergence could also be investigated through evolutionary time to determine whether the divergence observed is a transient intermediate condition that may subsequently be expressed, following further selection, as evidence for adaptation.

It is also possible that the divergent patterns of development and larval survival in the two regimes are influenced by the benefits of achieving specific “nutritional targets” (Simpson and Raubenheimer 1993). For example, if specific nutritional cues coselect sets of traits that together influence larval development then, once established, perturbation of such systems by proximate dietary cues may be difficult (Davidowitz et al. 2005). Again, it would be interesting to measure the specific benefits of such systems.

As a generalist, the medfly displays remarkable flexibility in development according to nutritional availability and is capable of changing larval development rates to maximize survival (Krainacker et al. 1987; Gasperi et al. 2002; Nash and Chapman 2014). Our experiments indicated that over successive generations dietary conditions experienced during development have significant and lasting effects on adult fitness. This is of importance for understanding of adaptive radiation in medfly (Gasperi et al., 2002; Karsten et al., 2015).

We discovered a mix of significant divergence in some traits such as development time and the maintenance of plasticity in adult behaviors in response to dietary selection. While larval development rate appeared to have diverged between selection regimes, the response in adult body mass showed strong evidence for local adaptation. These results may help to explain the previous observations of altered developmental physiology between isolated populations of medfly as a result of nutritional adaptation rather than alternative selection pressures or genetic drift (Diamantidis et al. 2011). The results provided no evidence that courtship behaviors showed evolved responses. Activity in courtship strongly reflected the nutrition available during larval development, suggesting an important role for larval‐adult nutritional carryover (Yuval et al. 1998; Kaspi et al. 2002).

Collectively the findings suggest great potential for further study of the relative role of plastic responses and adaptation in responses to novel selection pressures in the medfly. Such studies may enable us to determine which physiological traits remain most resistant to selection and/or display greatest plasticity. In doing so we may discover the features that distinguish globally invasive pests such as medfly from other pest species. Furthermore, we may be able to more accurately gauge the relative risk of invasiveness for a population when we are better able to identify and predict changing physiological responses of organisms to new dietary environments.

Associate Editor: T. Flatt

Handling Editor: M. Servedio

## Supporting information


**Table S1A** Generalised linear mixed model of the proportion of medfly eggs that survived to adult eclosion when reared on the ASG and Starch selection regimes across multiple generations
**Table S1B** Generalised linear mixed model of the proportion of medfly eggs that survived to adult eclosion when reared on the ASG and Starch selection regimes between generations 3‐5
**Table S1C** Generalised linear mixed model of the proportion of medfly eggs that survived to adult eclosion when reared on the ASG and Starch selection regimes at generation 30
**Table S1D** Generalised linear mixed model of the proportion of medfly eggs that survived to pupation when reared on the ASG and Starch selection regimes across multiple generations
**Table S1E** Generalised linear mixed model of the proportion of medfly eggs that survived to pupation when reared on the ASG and Starch selection regimes between generations 3‐5
**Table S1F** Generalised linear mixed model of the proportion of medfly eggs that survived to pupation when reared on the ASG and Starch selection regimes at generation 30
**Table S1G** Generalised linear mixed model of the proportion of medfly pupae that survived to adult eclosion when reared on the ASG and Starch selection regimes across multiple generations
**Table S1G** Generalised linear mixed model of the proportion of medfly pupae that survived to adult eclosion when reared on the ASG and Starch selection regimes across multiple generations
**Table S1I** Generalised linear mixed model of the proportion of medfly pupae that survived to adult eclosion when reared on the ASG and Starch selection regimes at generation 30
**Table S2A** Linear mixed model of the development time for medfly eggs that survived to adult eclosion when reared on the ASG and Starch selection regimes across multiple generations
**Table S2B** Linear mixed model of the development time for medfly eggs that survived to adult eclosion when reared on the ASG and Starch selection regimes between generations 3‐5
**Table S2C** Linear mixed model of the development time for medfly eggs that survived to adult eclosion when reared on the ASG and Starch selection regimes at generation 30
**Table S2D** Linear mixed model of the development time for medfly eggs that survived to pupation when reared on the ASG and Starch selection regimes across multiple generations
**Table S2E** Linear mixed model of the development time for medfly eggs that survived to pupation when reared on the ASG and Starch selection regimes between generations 3‐5
**Table S2F** Linear mixed model of the development time for medfly eggs that survived to pupation when reared on the ASG and Starch selection regimes at generation 30
**Table S2G** Linear mixed model of the development time for medfly pupae that survived to adult eclosion when reared on the ASG and Starch selection regimes across multiple generations
**Table S2H** Linear mixed model of the development time for medfly pupae that survived to adult eclosion when reared on the ASG and Starch selection regimes at generation 30
**Table S3A** Linear mixed model of adult male bodyweight across multiple generations
**Table S3B** Linear mixed model of adult female bodyweight across multiple generations
**Table S3C** Linear mixed model of adult male bodyweight between generations 3‐5
**Table S3D** Linear mixed model of adult female bodyweight between generations 3‐5
**Table S3E** Linear mixed model of adult male bodyweight at generation 30
**Table S3F** Linear mixed model of adult female bodyweight at generation 30
**Table S4A** Generalized linear mixed model of courtship latency for males and females reared ‘on diet’ at Generation 29.
**Table S4B** Generalized linear mixed model of copulation latency for males and females reared ‘on diet’ at Generation 29
**Table S5A** Generalised linear mixed model for males reared ‘on diet’ at Generation 29 for bout frequency of continuous wing vibration behaviour
**Table S5B** Generalised linear mixed model for males reared ‘on diet’ at Generation 29 for bout frequency of intermittent wing vibration behaviour
**Table S5C** Generalised linear mixed model for males reared ‘on diet’ at Generation 29 for bout frequency of head rocking behaviour
**Table S5D** Generalised linear mixed model for males reared ‘on diet’ at Generation 29 for bout frequency of gland extrusion behaviour
**Table S5E** Generalised linear mixed model for males reared ‘on diet’ at Generation 30 for bout frequency of continuous wing vibration behaviour
**Table S5F** Generalised linear mixed model for males reared ‘on diet’ at Generation 30 for bout frequency of intermittent wing vibration behaviour
**Table S5G** Generalised linear mixed model for males reared ‘on diet’ at Generation 30 for bout frequency of head rocking behaviour
**Table S5H** Generalised linear mixed model for males reared ‘on diet’ at Generation 30 for bout frequency of gland extrusion behaviour
