# Supplementary material for: Adaptation to divergent larval diets in the medfly, Ceratitis capitata
Source: Evolution. 2016 Nov 24;71(2):289–303. doi: 10.1111/evo.13113 (PMC5324619; doi:10.1111/evo.13113)
Supplement: Supplementary file 1 — Table S1A Generalised linear mixed model of the proportion of medfly eggs that survived to adult eclosion when reared on the ASG and Starch selection regimes across multiple generations Table S1B Generalised linear mixed model of the proportion of medfly eggs that survived to adult eclosion when reared on the ASG and Starch selection regimes between generations 3‐5 Table S1C Generalised linear mixed model of the proportion of medfly eggs that survived to adult eclosion when reared on the ASG and Starch selection regimes at generation 30 Table S1D Generalised linear mixed model of the proportion of medfly eggs that survived to pupation when reared on the ASG and Starch selection regimes across multiple generations Table S1E Generalised linear mixed model of the proportion of medfly eggs that survived to pupation when reared on the ASG and Starch selection regimes between generations 3‐5 Table S1F Generalised linear mixed model of the proportion of medfly eggs that survived to pupation when reared on the ASG and Starch selection regimes at generation 30 Table S1G Generalised linear mixed model of the proportion of medfly pupae that survived to adult eclosion when reared on the ASG and Starch selection regimes across multiple generations Table S1G Generalised linear mixed model of the proportion of medfly pupae that survived to adult eclosion when reared on the ASG and Starch selection regimes across multiple generations Table S1I Generalised linear mixed model of the proportion of medfly pupae that survived to adult eclosion when reared on the ASG and Starch selection regimes at generation 30 Table S2A Linear mixed model of the development time for medfly eggs that survived to adult eclosion when reared on the ASG and Starch selection regimes across multiple generations Table S2B Linear mixed model of the development time for medfly eggs that survived to adult eclosion when reared on the ASG and Starch selection regimes between generations 3‐5 Table S2C Linear mixed m [file EVO-71-289-s001.docx]

# Adaptation to divergent larval diets in the medfly, *Ceratitis capitata*

## Philip T. Leftwich*, William J. Nash*, Lucy A. Friend, and Tracey Chapman^

### *School of Biological Sciences, University of East Anglia, Norwich Research Park, Norwich, NR4 7TJ, UK*

*Joint authors

*^E-mail: tracey.chapman@uea.ac.uk*

**SUPPLEMENTARY INFORMATION**

## SUPPLEMENTARY TABLES

#### SECTION 1

The proportion of medfly eggs that survived to adult eclosion when reared on the ASG and Starch selection regimes was examined using generalized linear mixed models with a binomial error distribution, and observation level random effects were employed to account for overdispersion. The minimal model in each analysis was selected by sequential likelihood ratio testing and this is presented in the tables below. Data from the early and late generations were analysed together with generation, selection regime and proximate larval diet as fixed effects with replicate lines nested as random effects within selection regime (Table S1A), this dataset was then divided to compare the dietary responses for early vs. late generations (Tables S1B,C). This procedure was also repeated for larval survival (Tables S1D,E,F) and pupal survival (Tables S1G,H,I).

#### Table S1A

Generalised linear mixed model of the proportion of medfly eggs that survived to adult eclosion when reared on the ASG and Starch selection regimes across multiple generations

| Fixed effects | Estimate (SE) | z value | p-value |
| --- | --- | --- | --- |
| Intercept | -0.06(0.09) | -0.64 | 0.523 |
| Generation(Late) | -0.55(0.11) | -5.12 | <0.001 |
| Regime(S) | 0.05(0.13) | 0.39 | 0.693 |
| Generation(Late) * Regime(S) | 0.97(0.15) | 6.45 | <0.001 |
| Random effects | **Variance (SD)** |  |  |
| Obs | 0.3(0.55) |  |  |
| Line * Regime | 0.01(0.1) |  |  |
| Regime | 0 |  |  |

#### Table S1B

Generalised linear mixed model of the proportion of medfly eggs that survived to adult eclosion when reared on the ASG and Starch selection regimes between generations 3-5

| Fixed effects | Estimate (SE) | z value | p-value |
| --- | --- | --- | --- |
| Intercept | 0.14(0.11) | 1.32 | 0.188 |
| Regime(S) | -0.19(0.15) | -1.21 | 0.23 |
| Diet(S) | -0.41(0.1) | -4.15 | <0.001 |
| Regime(S) * Diet(S) | 0.48(0.14) | 3.44 | 0.001 |
| Random effects | **Variance (SD)** |  |  |
| Obs | 0.12(0.35) |  |  |
| Line * Regime | 0.002(0.14) |  |  |
| Regime | <0.001(<0.001) |  |  |

#### Table S1C

Generalised linear mixed model of the proportion of medfly eggs that survived to adult eclosion when reared on the ASG and Starch selection regimes at generation 30

| Fixed effects | | Estimate (SE) | z value | p-value |
| --- | --- | --- | --- | --- |
| Intercept | -0.62(0.1) | | -6.38 | <0.001 |
| Regime(S) | 1.03(0.13) | | 7.58 | <0.001 |
| Random effects | **Variance (SD)** | |  |  |
| Obs | 0.48(0.69) | |  |  |
| Line * Regime | 0 | |  |  |
| Regime | 0 | |  |  |

#### Table S1D

Generalised linear mixed model of the proportion of medfly eggs that survived to pupation when reared on the ASG and Starch selection regimes across multiple generations

| Fixed effects | Estimate (SE) | z value | p-value |
| --- | --- | --- | --- |
| Intercept | 0.13(0.11) | 1.19 | 0.235 |
| Generation(Late) | -0.7(0.14) | -4.94 | <0.001 |
| Regime(S) | 0.1(0.13) | 0.78 | 0.439 |
| Diet(S) | -0.13(0.11) | -1.19 | 0.235 |
| Generation(Late) * Regime(S) | 1.07(0.16) | 6.59 | <0.001 |
| Generation(Late) * Diet(S) | 0.4(0.16) | 2.47 | 0.014 |
| Random effects | **Variance (SD)** |  |  |
| Obs | 0.35(0.59) |  |  |
| Line * Regime | 0.01(0.08) |  |  |
| Regime | 0 |  |  |

#### Table S1E

Generalised linear mixed model of the proportion of medfly eggs that survived to pupation when reared on the ASG and Starch selection regimes between generations 3-5

| Fixed effects | Estimate (SE) | z value | p-value |
| --- | --- | --- | --- |
| Intercept | 0.26(0.11) | 2.39 | 0.017 |
| Regime(S) | -0.17(0.15) | -1.1 | 0.273 |
| Diet(S) | -0.39(0.1) | -3.97 | <0.001 |
| Regime(S) * Diet(S) | 0.54(0.14) | 3.81 | <0.001 |
| Random effects | **Variance (SD)** |  |  |
| Obs | 0.13(0.36) |  |  |
| Line * Regime | 0.02(0.13) |  |  |
| Regime | 0 |  |  |

#### Table S1F

Generalised linear mixed model of the proportion of medfly eggs that survived to pupation when reared on the ASG and Starch selection regimes at generation 30

| Fixed effects | Estimate (SE) | z value | p-value |
| --- | --- | --- | --- |
| Intercept | -0.57(0.13) | -4.32 | <0.001 |
| Regime(S) | 1.19(0.15) | 7.68 | <0.001 |
| Diet(S) | 0.27(0.15) | 1.75 | 0.08 |
| Random effects | **Variance (SD)** |  |  |
| Obs | 0.6(0.77) |  |  |
| Line * Regime | <0.001(<0.001) |  |  |
| Regime | <0.001(<0.001) |  |  |

#### Table S1G

Generalised linear mixed model of the proportion of medfly pupae that survived to adult eclosion when reared on the ASG and Starch selection regimes across multiple generations

| Fixed effects | Estimate (SE) | z value | p-value |
| --- | --- | --- | --- |
| Intercept | 3.22(0.18) | 17.31 | <0.001 |
| Generation(Late) | -0.09(0.18) | -0.51 | 0.61 |
| Diet(S) | -0.44(0.13) | -3.19 | 0.001 |
| Regime(S) | -0.39(0.23) | -1.64 | 0.1 |
| Generation(Late) * Regime(S) | 0.44(0.2) | 2.2 | 0.03 |
| Generation(Late) * Diet(S) | -0.74(0.2) | -3.73 | <0.001 |
| Random effects | **Variance (SD)** |  |  |
| Obs | 0.37(<0.001) |  |  |
| Line * Regime | 0.06(<0.001) |  |  |
| Regime | <0.001(<0.001) |  |  |

#### Table S1H

Generalised linear mixed model of the proportion of medfly pupae that survived to adult eclosion when reared on the ASG and Starch selection regimes at generations 3-5

| Fixed effects | Estimate (SE) | z value | p-value |
| --- | --- | --- | --- |
| Intercept | 2.96(0.15) | 19.11 | <0.001 |
| Diet(S) | -0.39(0.1) | -3.82 | <0.001 |
| Random effects | **Variance (SD)** |  |  |
| Obs | 0.14(0.38) |  |  |
| Line * Regime | 0.11(0.33) |  |  |
| Regime | <0.001(<0.001) |  |  |

#### Table S1I

Generalised linear mixed model of the proportion of medfly pupae that survived to adult eclosion when reared on the ASG and Starch selection regimes at generation 30

| Fixed effects | | Estimate (SE) | z value | p-value | |
| --- | --- | --- | --- | --- | --- |
| Intercept | 3.22(0.15) | | 20.73 | <0.001 |  |
| Diet(S) | -1.23(0.17) | | -6.96 | <0.001 |  |
| Random effects | **Variance (SD)** | |  |  |  |
| Obs | 0.62(0.79) | |  |  |  |
| Line * Regime | 0.04(0.19) | |  |  |  |
| Regime | 0 | |  |  |  |

#### SECTION 2

The development time for medfly eggs that survived to adult eclosion when reared on the ASG and Starch selection regimes was examined using linear mixed models. The minimal model in each analysis was selected by sequential likelihood ratio testing and this is presented in the tables below. Data from the early and late generations were analysed together with generation, selection regime and proximate larval diet as fixed effects with replicate lines nested as random effects within selection regime (Table S2A), this dataset was then divided to compare the dietary responses for early vs. late generations (Tables S2B,C). This procedure was also repeated for larval development time (Tables S2D,E,F) and pupal development time (Tables S2G,H).

#### Table S2A

Linear mixed model of the development time for medfly eggs that survived to adult eclosion when reared on the ASG and Starch selection regimes across multiple generations

| Fixed effects | Estimate (SE) | df | t value | p-value |
| --- | --- | --- | --- | --- |
| Intercept | 23.59(0.14) | 228 | 161.32 | <0.001 |
| Generation(Late) | -3.66(0.17) |  | -21.26 | <0.001 |
| Regime(S) | -0.49(0.17) |  | -2.85 | 0.005 |
| Diet(S) | -0.76(0.21) |  | -3.69 | <0.001 |
| Generation(Late) * Diet(S) | 1.9(0.24) |  | 7.8 | <0.001 |
| Diet(S) * Regime(S) | -0.59(0.24) |  | -2.41 | 0.017 |
| Random effects | **Variance (SD)** |  |  |  |
| Line * Regime | 0 |  |  |  |
| Regime | 0 |  |  |  |
| Residual | 0.84(0.92) |  |  |  |

#### Table S2B

Linear mixed model of the development time for medfly eggs that survived to adult eclosion when reared on the ASG and Starch selection regimes between generations 3-5

| Fixed effects | Estimate (SE) | df | t value | p-value |
| --- | --- | --- | --- | --- |
| Intercept | 23.44(0.14) | 120 | 166.28 | <0.001 |
| Regime(S) | -0.19(0.19) |  | -0.95 | 0.34 |
| Diet(S) | -0.54(0.19) |  | -2.71 | 0.008 |
| Diet(S) * Regime(S) | -1.03(0.28) |  | -3.65 | <0.001 |
| Random effects | **Variance (SD)** |  |  |  |
| Line * Regime | 0 |  |  |  |
| Regime | 0 |  |  |  |
| Residual | 0.59(0.77) |  |  |  |

#### Table S2C

Linear mixed model of the development time for medfly eggs that survived to adult eclosion when reared on the ASG and Starch selection regimes at generation 30

| Fixed effects | Estimate (SE) | df | t value | p-value |
| --- | --- | --- | --- | --- |
| Intercept | 20.12(0.17) | 108 | 116.16 | <0.001 |
| Regime(S) | -0.87(0.2) |  | -4.34 | <0.001 |
| Diet(S) | 0.84(0.2) |  | 4.21 | <0.001 |
| Random effects | **Variance (SD)** |  |  |  |
| Line * Regime | 0 |  |  |  |
| Regime | 0 |  |  |  |
| Residual | 1.08(1.04) |  |  |  |

#### Table S2D

Linear mixed model of the development time for medfly eggs that survived to pupation when reared on the ASG and Starch selection regimes across multiple generations

| Fixed effects | Estimate (SE) | df | t value | p-value |
| --- | --- | --- | --- | --- |
| Intercept | 15.57(0.14) | 228 | 105.15 | <0.001 |
| Generation(Late) | -3.84(0.19) |  | -19.38 | <0.001 |
| Regime(S) | 0.39(0.19) |  | -1.98 | 0.049 |
| Diet(S) | -0.85(0.19) |  | -4.38 | <0.001 |
| Generation(Late) * Regime(S) | 0.36(0.23) |  | 1.58 | 0.11 |
| Generation(Late) * Diet(S) | 1.85(0.23) |  | 8.1 | <0.001 |
| Diet(S) * Regime(S) | -0.63(0.23 |  | -2.76 | 0.006 |
| Random effects | **Variance (SD)** |  |  |  |
| Line * Regime | <0.001(<0.001) |  |  |  |
| Regime | <0.001(<0.001) |  |  |  |
| Residual | <0.001(<0.001) |  |  |  |

#### Table S2E

Linear mixed model of the development time for medfly eggs that survived to pupation when reared on the ASG and Starch selection regimes between generations 3-5

| Fixed effects | Estimate (SE) | df | t value | p-value |
| --- | --- | --- | --- | --- |
| Intercept | 15.72(0.14) | 120 | 107.51 | <0.001 |
| Regime(S) | -0.07(0.16) |  | -4.16 | <0.001 |
| Diet(S) | -1.17(0.17) |  | -6.92 | <0.001 |
| Random effects | **Variance (SD)** |  |  |  |
| Line * Regime | 0 |  |  |  |
| Regime | 0 |  |  |  |
| Residual | 0.86(0.92) |  |  |  |

#### Table S2F

Linear mixed model of the development time for medfly eggs that survived to pupation when reared on the ASG and Starch selection regimes at generation 30

| Fixed effects | Estimate (SE) | df | t value | p-value |
| --- | --- | --- | --- | --- |
| Intercept | 11.71(0.14) | 102 | 81.62 | <0.001 |
| Diet(S) | 0.69(0.16) |  | 4.36 | <0.001 |
| Random effects | **Variance (SD)** |  |  |  |
| Line * Regime | 0.03(0.15) |  |  |  |
| Regime | 0.008(0.09) |  |  |  |
| Residual | 0.67(0.82) |  |  |  |

#### Table S2G

Linear mixed model of the development time for medfly pupae that survived to adult eclosion when reared on the ASG and Starch selection regimes across multiple generations

| Fixed effects | | Estimate (SE) | df | t value | p-value |
| --- | --- | --- | --- | --- | --- |
| Intercept | 7.96(0.08) | | 228 | 97.95 | <0.001 |
| Generation(Late) | 0.34(0.11) | |  | 3.26 | 0.001 |
| Regime(S) | -0.003(0.1) | |  | -0.03 | 0.97 |
| Diet(S) | 0.14(0.07) | |  | 1.96 | 0.05 |
| Diet (S)* Regime(S) | -0.54(0.14) | |  | -3.69 | <0.001 |
| Random effects | **Variance (SD)** | |  |  |  |
| Line * Regime | 0 | |  |  |  |
| Regime | 0 | |  |  |  |
| Residual | 0.31(0.56) | |  |  |  |

#### Table S2H

Linear mixed model of the development time for medfly pupae that survived to adult eclosion when reared on the ASG and Starch selection regimes at generation 30

| Fixed effects | | Estimate (SE) | df | t value | p-value |
| --- | --- | --- | --- | --- | --- |
| Intercept | 8.37(0.08) | | 108 | 107.55 | <0.001 |
| Regime(S) | -0.55 | |  | -5.01 | <0.001 |
| Random effects | **Variance (SD)** | |  |  |  |
| Line * Regime | 0 | |  |  |  |
| Regime | 0 | |  |  |  |
| Residual | 0.33(0.57) | |  |  |  |

**SECTION 3**

The adult body mass for medfly reared on the ASG and Starch selection regimes was examined using linear mixed models. The minimal model in each analysis was selected by sequential likelihood ratio testing and this is presented in the tables below. Data from the early and late generations were analysed together with generation, selection regime and proximate larval diet as fixed effects with replicate lines nested as random effects within selection regime for males and females (Tables S3A,B), this dataset was then divided to compare the dietary responses for early (Tables S3C,D) vs. late generations (Tables S3E,F).

#### Table S3A

Linear mixed model of adult male bodyweight across multiple generations

| Fixed effects | | Estimate (SE) | df | t value | p-value |
| --- | --- | --- | --- | --- | --- |
| Intercept | 1.25(0.03) | | 228 | 47.1 | <0.001 |
| Generation(Late) | 0.37(0.04) | |  | 9.67 | <0.001 |
| Regime(S) | -0.02(0.03) | |  | -0.71 | 0.48 |
| Diet(S) | -0.02(0.03) | |  | -0.62 | 0.53 |
| Gen(Late) * Regime(S) | -0.08(0.05) | |  | -1.42 | 0.16 |
| Gen(Late) * Diet(S) | -0.16(0.05) | |  | -3.04 | 0.003 |
| Regime(S) * Diet(S) | -0.18(0.05) | |  | -3.34 | <0.001 |
| Gen(Late) * Regime(S) * Diet(S) | 0.48(0.08) | |  | 6.25 | <0.001 |
| Random effects | **Variance (SD)** | |  |  |  |
| Line * Regime | 0 | |  |  |  |
| Regime | 0 | |  |  |  |
| Residual | 0.02(0.15) | |  |  |  |

#### Table S3B

Linear mixed model of adult female bodyweight across multiple generations

| Fixed effects | | Estimate (SE) | df | t value | p-value |
| --- | --- | --- | --- | --- | --- |
| Intercept | 1.39(0.02) | | 228 | 61.4 | <0.001 |
| Generation(Late) | 0.36(0.03) | |  | 12.3 | <0.001 |
| Regime(S) | -0.07(0.02) | |  | -3.27 | 0.001 |
| Diet(S) | -0.12(0.03) | |  | -4.33 | <0.001 |
| Gen(Late) * Diet(S) | 0.17(0.04) | |  | 4.19 | <0.001 |
| Random effects | **Variance (SD)** | |  |  |  |
| Line * Regime | 0 | |  |  |  |
| Regime | 0 | |  |  |  |
| Residual | 0.02(0.15) | |  |  |  |

#### Table S3C

Linear mixed model of adult male bodyweight between generations 3-5

| Fixed effects | | Estimate (SE) | df | t value | p-value |
| --- | --- | --- | --- | --- | --- |
| Intercept | 1.25(0.03) | | 120 | 42.32 | <0.001 |
| Regime(S) | -0.03(0.04) | |  | -0.06 | 0.52 |
| Diet(S) | -0.02(0.04) | |  | -0.56 | 0.58 |
| Regime(S) * Diet(S) | -0.18(0.06) | |  | -3 | 0.003 |
| Random effects | **Variance (SD)** | |  |  |  |
| Line * Regime | 0 | |  |  |  |
| Regime | 0 | |  |  |  |
| Residual | 0.03(0.16) | |  |  |  |

#### Table S3D

Linear mixed model of adult female bodyweight between generations 3-5

| Fixed effects | | Estimate (SE) | df | t value | p-value |
| --- | --- | --- | --- | --- | --- |
| Intercept | 1.42(0.03) | | 120 | 52.28 | <0.001 |
| Regime(S) | -0.14(0.03) | |  | -4.35 | <0.001 |
| Diet(S) | -0.12 | |  | -3.92 | <0.001 |
| Random effects | **Variance (SD)** | |  |  |  |
| Line * Regime | 0 | |  |  |  |
| Regime | 0 | |  |  |  |
| Residual | 0.03(0.17) | |  |  |  |

#### Table S3E

Linear mixed model of adult male bodyweight at generation 30

| Fixed effects | | Estimate (SE) | df | t value | p-value |
| --- | --- | --- | --- | --- | --- |
| Intercept | 1.62(0.02) | | 108 | 67.68 | <0.001 |
| Regime(S) | -0.1(0.03) | |  | -3.07 | 0.003 |
| Diet(S) | -0.19(0.03) | |  | -5.59 | <0.001 |
| Regime(S) * Diet(S) | 0.3(0.05) | |  | 6.35 | <0.001 |
| Random effects | **Variance (SD)** | |  |  |  |
| Line * Regime | <0.001(<0.001) | |  |  |  |
| Regime | 0 | |  |  |  |
| Residual | 0.02(0.12) | |  |  |  |

#### Table S3F

Linear mixed model of adult female bodyweight at generation 30

| Fixed effects | | Estimate (SE) | df | t value | p-value |
| --- | --- | --- | --- | --- | --- |
| Intercept | 1.71(0.02) | | 108 | 100.7 | <0.001 |
| Diet(S) | 0.05(0.02) | |  | 2.08 | 0.04 |
| Random effects | **Variance (SD)** | |  |  |  |
| Line * Regime | 0 | |  |  |  |
| Regime | 0 | |  |  |  |
| Residual | 0.02(0.12) | |  |  |  |

#### SECTION 4

Latency to the initiation of courtship and copulation for no choice mating tests was examined using generalised linear mixed models fitted for the time in milliseconds from the initiation of the mating test to the first occurrence of one of the four courtship behaviours and observation of a settled copulation; observation level random effects were employed to account for overdispersion. The minimal model in each analysis was selected by sequential likelihood ratio testing and this is presented in the tables below. Data is from males reared ‘on diet’ and paired with females ‘on diet’ and was collected at Gen 29.

#### Table S4A

Generalized linear mixed model of courtship latency for males and females reared ‘on diet’ at Generation 29.

| Fixed effects | | Estimate (SE) | z value | p-value |
| --- | --- | --- | --- | --- |
| Intercept | 11.99(0.17) | | 71.9 | <0.001 |
| Male Diet | 0.64(0.25) | | 2.53 | 0.012 |
| Female Diet | 0.65(0.21) | | 3.15 | 0.002 |
| Random effects | **Variance (SD)** | |  |  |
| Obs | 1.21(1.1) | |  |  |
| Line*Male Diet | 0.02(0.14) | |  |  |
| Male Diet | 0 | |  |  |

#### Table S4B

Generalized linear mixed model of copulation latency for males and females reared ‘on diet’ at Generation 29

| Fixed effects | | Estimate (SE) | z value | p-value |
| --- | --- | --- | --- | --- |
| Intercept | 13.2(0.08) | | 173.5 | >0.001 |
| Male Diet | 0.42(0.14) | | 3 | 0.003 |
| Random effects | **Variance (SD)** | |  |  |
| Obs | 0.49(0.7) | |  |  |
| Line*Male Diet | 2.67 e-15(4.76 e-8) | |  |  |
| Male Diet | 0 | |  |  |

#### SECTION 5

Generalise linear mixed models were fitted for bout frequency (bf), measured as the number of times a behaviour occurred from the start of filming to the occurrence of copulation, observation level random effects were employed to account for overdispersion. The minimal model in each analysis was selected by sequential likelihood ratio testing and this is presented in the tables below. For tables S5A-D, data are from males reared ‘on diet’ and paired with females ‘on diet’ and was collected at Gen 29. For tables S5E-H, data are from males reared on the ‘opposite diet’ and paired with females ‘on diet’

#### Table S5A

Generalised linear mixed model for males reared ‘on diet’ at Generation 29 for bout frequency of continuous wing vibration behaviour

| Fixed effects | | Estimate (SE) | z value | p-value |
| --- | --- | --- | --- | --- |
| Intercept | -11.91(0.15) | | -79.91 | <0.001 |
| Male diet(S) | -0.46(0.23) | | -2.05 | 0.04 |
| Female diet(S) | -0.51(0.21) | | -2.42 | 0.016 |
| Random effects | **Variance (SD)** | |  |  |
| Obs | 0.88(0.94) | |  |  |
| Line * Male Diet | 0 | |  |  |
| Male Diet | 0 | |  |  |

#### Table S5B

Generalised linear mixed model for males reared ‘on diet’ at Generation 29 for bout frequency of intermittent wing vibration behaviour

| Fixed effects | | Estimate (SE) | z value | p-value |
| --- | --- | --- | --- | --- |
| Intercept | -12.24(0.15) | | -84.23 | <0.001 |
| Male diet(S) | -0.61(0.22) | | -2.73 | 0.006 |
| Female diet(S) | -0.55(0.21) | | -2.65 | 0.008 |
| Random effects | **Variance (SD)** | |  |  |
| Obs | 0.72(0.85) | |  |  |
| Line * Male Diet | 0 | |  |  |
| Male Diet | 0 | |  |  |

#### Table S5C

Generalised linear mixed model for males reared ‘on diet’ at Generation 29 for bout frequency of head rocking behaviour

| Fixed effects | | Estimate (SE) | z value | p-value |
| --- | --- | --- | --- | --- |
| Intercept | -11.83(0.16) | | -75.1 | <0.001 |
| Male diet(S) | -1.36(0.31) | | -4.38 | <0.001 |
| Female diet(S) | -0.99(0.26) | | -3.84 | <0.001 |
| Male diet(S) * Female diet(S) | 0.88(0.51) | | 1.74 | 0.081 |
| Random effects | **Variance (SD)** | |  |  |
| Obs | 0.91(0.95) | |  |  |
| Line * Male Diet | 0 | |  |  |
| Male Diet | 0 | |  |  |

#### Table S5D

Generalised linear mixed model for males reared ‘on diet’ at Generation 29 for bout frequency of gland extrusion behaviour

| Fixed effects | | Estimate (SE) | z value | p-value |
| --- | --- | --- | --- | --- |
| Intercept | -13.93(0.26) | | -53.45 | <0.001 |
| Male diet(S) | 0.77(0.37) | | 2.07 | 0.038 |
| Random effects | **Variance (SD)** | |  |  |
| Obs | 1.58(1.26) | |  |  |
| Line * Male Diet | 0.02(0.16) | |  |  |
| Male Diet | <0.001(<0.001) | |  |  |

#### Table S5E

Generalised linear mixed model for males reared ‘on diet’ at Generation 30 for bout frequency of continuous wing vibration behaviour

| Fixed effects | | Estimate (SE) | z value | p-value |
| --- | --- | --- | --- | --- |
| Intercept | -11.56(0.22) | | -51.77 | <0.001 |
| Female diet(S) | -1.51(0.29) | | -5.144 | <0.001 |
| Random effects | **Variance (SD)** | |  |  |
| Obs | 1.19(1.09) | |  |  |
| Line * Male Diet | 0 | |  |  |
| Male Diet | 0 | |  |  |

#### Table S5F

Generalised linear mixed model for males reared ‘on diet’ at Generation 30 for bout frequency of intermittent wing vibration behaviour

| Fixed effects | | Estimate (SE) | z value | p-value |
| --- | --- | --- | --- | --- |
| Intercept | -12.01(0.22) | | -55.08 | <0.001 |
| Female diet(S) | -1.52(0.27) | | -5.54 | <0.001 |
| Random effects | **Variance (SD)** | |  |  |
| Obs | 0.72(0.85) | |  |  |
| Line * Male Diet | 0.01(0.07) | |  |  |
| Male Diet | 0 | |  |  |

#### Table S5G

Generalised linear mixed model for males reared ‘on diet’ at Generation 30 for bout frequency of head rocking behaviour

| Fixed effects | | Estimate (SE) | z value | p-value |
| --- | --- | --- | --- | --- |
| Intercept | -11.9(0.22) | | -53.21 | <0.001 |
| Female diet(S) | -1.18(0.22) | | -5.39 | <0.001 |
| Random effects | **Variance (SD)** | |  |  |
| Obs | 0.48(0.69) | |  |  |
| Line * Male Diet | 0.13(0.36) | |  |  |
| Male Diet | 0 | |  |  |

#### Table S5H

Generalised linear mixed model for males reared ‘on diet’ at Generation 30 for bout frequency of gland extrusion behaviour

| Fixed effects | | Estimate (SE) | z value | p-value |
| --- | --- | --- | --- | --- |
| Intercept | -13.17(0.35) | | -37.58 | <0.001 |
| Male diet(S) | 0.58(0.34) | | 1.7 | 0.09 |
| Female diet(S) | -0.94(0.32) | | -2.92 | 0.004 |
| Random effects | **Variance (SD)** | |  |  |
| Obs | 1.13(1.07) | |  |  |
| Line * Male Diet | <0.001(<0.001) | |  |  |
| Male Diet | <0.001(<0.001) | |  |  |
